# Surface-Enhanced Raman Scattering in Silver-Coated Suspended-Core Fiber

**DOI:** 10.3390/s24010160

**Published:** 2023-12-27

**Authors:** Yangyang Xu, Xian Zhang, Xiao-Song Zhu, Yi-Wei Shi

**Affiliations:** 1 School of Information Science and Engineering, Fudan University, 220 Handan Rd, Shanghai 200433, China; 20110720067@fudan.edu.cn (Y.X.); 18110720057@fudan.edu.cn (X.Z.); ywshi@fudan.edu.cn (Y.-W.S.); 2 Yiwu Research Institute of Fudan University, Chengbei Road, Yiwu City 322000, China; 3 Key Laboratory for Information Science of Electromagnetic Waves (MoE), Fudan University, 220 Handan Rd, Shanghai 200433, China

**Keywords:** surface-enhanced Raman scattering, large-core suspended-core fiber, silver film, 4-mercaptophenylboronic acid

## Abstract

In this paper, the silver-coated large-core suspended-core fiber (LSCF) probe was fabricated by the dynamic chemical liquid phase deposition method for surface-enhanced Raman scattering (SERS) sensing. The 4-mercaptophenylboronic acid (4-MPBA) monolayer was assembled in the LSCF as the recognition monolayer. Taking advantage of the appropriate core size of the LSCF, a custom-made Y-type optical fiber patch cable was utilized to connect the semiconductor laser, Raman spectrometer, and the proposed fiber SERS probe. The SERS signal is propagated in the silver-coated air channels, which can effectively reduce the Raman and fluorescence background of the silica core. Experiments were performed to measure the Raman scattering spectra of the 4-MPBA in the silver-coated LSCF in a non-enhanced and enhanced case**.** The experiment results showed that the Raman signal strength was enhanced more than 6 times by the surface plasmon resonance compared with the non-enhanced case. The proposed LSCF for SERS sensing technology provides huge research value for the fiber SERS probes in biomedicine and environmental science. The combination of SERS and microstructured optical fibers offers a potential approach for SERS detection

## 1. Introduction

Raman scattering is a photon–molecular interaction which provides “fingerprint” information on molecular vibration, rotation, and other low-wavenumber transitions of the investigated samples [1,2,3]. Photons of a laser are absorbed by the molecules and then reemitted. The wavenumber of the reemitted photons is shifted up or down compared with the original monochromatic wavenumber. The unique fingerprint-type signals allow for molecular identification. Due to its high chemical sensitivity, uncomplicated sample preparation, and excellent specificity, applications of the Raman spectroscopic technique have experienced a rapid development in decades [4]. However, the inherently Raman signal strength leads to serious challenges in Raman measurements, including trace concentration detection and real-time remote sensing.

Surface-enhanced Raman scattering (SERS) spectroscopy is a technology derived from Raman spectroscopy that detects trace amounts of chemicals and identifies them based on their unique vibrational properties. It is widely used in food safety, clinical medicine, environmental monitoring, biochemistry fields, and many more [5,6,7,8]. There is general agreement that SERS mechanisms includes the electromagnetic enhancement mechanism (EM) and the chemical enhancement (CE) mechanism [9,10,11]. The enhancement of the electromagnetic field, which is induced by surface plasmon polaritons (SPP), usually enhances the Raman signal strength over a wide frequency range. SPP is a collective oscillation of free electrons in the metal, which can be excited when appropriate light is incident on the surface. Furthermore, the surface plasmon resonance (SPR) technique is based on SPP excited at the metal–dielectric interface when a wave vector matching condition is satisfied [12]. SPR sensors have been widely applied in biosensing because of their ability to effectively detect the changes in the refractive index (RI) of the surrounding medium [13].

Traditional SERS substrates commonly have metallic nanostructures on rigid flat-based materials (e.g., silicon chips, glass slide) [14,15]. However, this kind of SERS substrate lack flexibility, which limits their large-scale application. To solve this problem, SERS substrates based on flexible materials have attracted widespread attention in recent years (such as papers, plastic polymers, and graphene oxide) [16,17,18] due to their advantages of low cost, flexibility, and ease of fabrication. For instance, Kim et al. developed a paper substrate for sensitive SERS detection in which the nanoparticles are directly synthesized within the paper without external processing [19]. Typically, manufacturing techniques such as electron beam lithography [20], focused-ion-beam (FIB) [21], and electrochemical deposition [22,23,24,25] are needed to fabricate well-ordered metal nanostructures on the substrates. However, these techniques may be time-consuming or expensive. Moreover, the light–analyte interaction area is limited by the spot size, which is only several microns in diameter. The poor reproducibility and lack of flexibility also limit the practical applications of planar SERS-active substrates.

With the rapid development of nanotechnology, the combined technology of SERS and optical fiber sensing emerges and attracts public attention [26,27,28]. This technology offers unique benefits over planar SERS substrates, such as good flexibility, compact structure, better molecular specificity, higher sensitivity, and remote sensing capability [28,29,30]. For example, A. Sánchez-Solí et al. demonstrated an optothermal-surface bubble-assisted printing method for depositing metallic nanoparticles on an optical fiber tip [31]. Chen et al. proposed a tapered optical-fiber-based SERS nanoprobe for Rhodamine 6G (R6G) aqueous solution detection [32]. Then, Xia et al. fabricated the spherically tipped fiber probe based on silver nanoparticlesto detect the R6G molecule [33]. Although they are easy to fabricate, the small number of SERS-active nanoparticles involved in the active region of the fiber end limits the sensitivity of such SERS probes, in which the optical fibers just serve as the waveguide to transmit the forward excitation light and collect the backward Raman scattering light. To overcome this hurdle, photonic crystal fibers (PCFs) with axially aligned air channels along the entire fiber length are explored to provide an excellent platform for SERS sensing. Owing to the long light–matter interaction length and extremely low sample volume, SERS-active PCF probes show great potential in trace-level biosensing in preclinical and clinical settings [34,35,36,37]. In hollow-core PCFs, both the excitation light and Raman scattering signal are confined in the liquid-filled central core by the photonic bandgap effect, resulting in improved light–analyte interaction along the fiber length. However, the narrow transmission window is spectrally shifted with the refractive index (RI) of the liquid filled in the hollow central core, which limits its applications in in vivo detection. Since the fiber is only partly filled by the capillary force, the weakly guided excitation light and SERS signal caused by the shifted bandgap would result in degraded sensor performance. Although liquid-core PCFs with a selectively filled hollow central core are proposed to enable a very large transmission window by total internal reflections, the process of sealing the cladding air channels with a fusion splicer is complicated and time-consuming. In the solid-core PCFs, the excitation light is propagated in the solid core usually made of silica by the index-guiding mechanism. The weak Raman signal of the analyte at low concentration may be masked by the strong Raman and fluorescence background of silica. SERS sensing with a solid-core PCF is realized through the interactions between the evanescent part of the guided light and the analyte. However, the power fraction of the evanescent field in the cladding air channels of conventional solid-core PCFs is less than 1%, which seriously limits the sensitivity of such SERS sensors. Khaing Oo et al. proposed a suspended-core PCF with enhanced evanescent field distribution in the cladding air channels, which is composed of a small silica core surrounded by a few large air channels for efficient liquid infiltration [38]. The fraction of evanescent light in the air holes is increased by simply reducing the core size, despite a poor coupling and collection efficiency of the excitation light and Raman scattering light.

For the aforementioned holey fiber structures, the metal nanoparticles are either immobilized on the inner surface of the air holes, or mixed with the sample solution and filled in the air channels. The SERS signal is accumulated along the fiber length, leading to higher sensitivity and lower detection limit. There is a trade-off between the enhancement in Raman signal and the additional loss, both induced by the metal nanoparticles. However, it is rather difficult to precisely control the coverage density of nanoparticles on the inner wall of tiny air channels with different shapes, which is necessary to fabricate SERS probes with good reproducibility and stability. By contrast, it is easier to deposit a uniform metal film with controllable thickness inside the holey fiber structures. A large-core suspended-core fiber (LSCF) is a subset of micro-structured optical fibers (MOFs). Due to their relatively simple structure and strong light–matter interaction, LSCFs offer excellent prospects for a multitude of scientific and technological applications [39]. They consist of a solid central core surrounded by an arrangement of several air holes, which run longitudinally along the length of the fiber [40]. Meanwhile, the fragile metal layer can be protected inside the air holes from contamination and corrosion. The accessibility to the air holes of LSCFs has also opened up the possibility for functionalization of the surfaces at the nanometer scales, in particular to impart the functionality of SERS in LSCF for sensing and detection. To the best of our knowledge, there has been no research about optical fiber sensors utilizing thin metal films for SERS sensing.

In this paper, we proposed an optical fiber SERS probe based on a silver-coated LSCF. A thin silver (Ag) layer is coated homogeneously along the length of the fiber on the inner surface of the air holes of the LSCF utilizing the liquid-phase deposition method. Taking advantage of the appropriate core size of the LSCF, a custom-made Y-type optical fiber patch cable was utilized to connect the semiconductor laser, Raman spectrometer, and the proposed fiber SERS probe. Thus, the system stability and simplicity are improved by omitting the complicated optical alignment equipment. The SERS sensing capability was investigated by coating the recognition monolayer on the silver film by the self-assembly monolayer (SAM) technique, while the air holes were filled with liquid with appropriate RI to adjust the resonance wavelength to around the laser wavelength. When the laser is transmitted through the fiber core, the SPR is excited and the electromagnetic field in the vicinity of the analyte monolayer is enhanced. The SERS signal is propagated in the silver-coated air channels instead of the silica core and collected by the Y-type optical fiber patch cable, which can effectively reduce the Raman and fluorescence background of the silica core. The long interaction length between the light and analyte enables the SERS signal to be accumulated along the entire fiber length. The result shows that Raman signal strength is enhanced more than 6 times compared with the non-enhanced case. Moreover, the proposed fiber probe can also serve as a SPR sensor by monitoring the resonance wavelength (RW) shift for analytes not suitable for SERS detection. Combining the optical fiber waveguide effect and SERS technology may provide a new platform for SERS sensing application.

## 2. Fabrication and Experimental Setup

### 2.1. Chemicals and Reagents

Glucose monohydrate (C_6_H_12_O_6_), silver nitrate (AgNO_3_, 99.8%), potassium hydroxide (KOH, 85.0%), and aqueous ammonia (~28%) were purchased from Sinopharm Chemical Reagent Co., Ltd. (Shanghai, China). Tin (ΙΙ) chloride anhydrous (SnCl_2_) and 4-MPBA (C_6_H_7_BO_2_S, 95%) were purchased from Aladdin Chemistry Co., Ltd. (Shanghai, China). Deionized (DI) water and absolute ethanol (99.8%) were used as solvents. All chemicals and reagents in the experiments were of analytical reagent grade and used without any further purification.

### 2.2. LSCF Configuration

**Figure 1:**
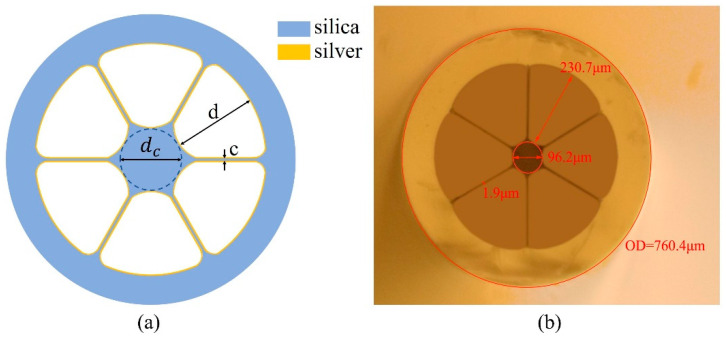
(**a**) Structure of the proposed silver-coated LSCF. (**b**) Microscope image of the cross section of the fabricated LSCF.

Figure 1a shows the structure of the proposed silver-coated LSCF with an outer diameter of approximately 760 μm. The geometry used here consists of six air holes surrounding a hexagonal silica core. The fabrication of this type of fiber relies on stacking six capillaries into a silica jacketing tube and drawing this arrangement into the fiber. A microscope image of the cross section of the fabricated LSCF is shown in Figure 1b. The core section is suspended by six struts of an approximate thickness of 2 μm, which guarantee the mechanical strength of the fiber. The core diameter *d_c_* is around 100 μm, which is defined as the diameter of the largest circle that can be inscribed in the core region. The light is constricted to guide in the solid core by the effective index contrast between the core and the surrounding air holes. A silver layer with thickness around 50 nm is coated on the inner surface of the air holes of the LSCF to support the excitation of the SPR phenomenon. Due to the large air holes with diameter around 230 μm (radial), the high-pressure condition is not needed during the silver layer deposition process. When appropriate light is transmitted in the solid silica core, SPP would be excited and electromagnetic field enhancement would occur at the interface between the analyte in the air holes and the silver layer adjacent to the silica core. Hence, the enhanced electromagnetic field caused by the SPR phenomenon would further enhance the Raman scattering signal of the analyte absorbed on the silver layer surface.

### 2.3. Preparation of the Ag-Coated LSCF

**Figure 2:**
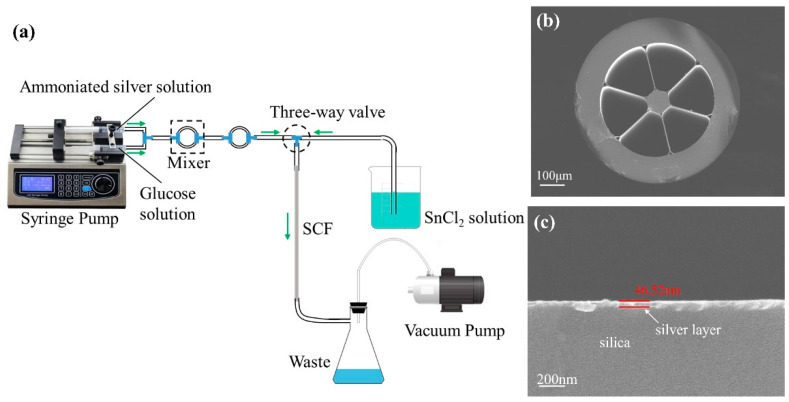
(**a**) Schematic diagram of the chemical liquid phase deposition method. (**b**) The whole cross-sectional SEM image of the silver-coated LSCF. (**c**) The cross-sectional SEM image of coated silver layer in the LSCF.

In brief, the improved liquid-phase deposition method was employed to coat a dense silver layer on the inner surface of the LSCF [40], as shown in Figure 2a. More importantly that the deposition time, the flow rate of solutions and temperature must be carefully controlled to obtain a uniform and smooth silver layer. Before deposition, the inner surface was sensitized with a 0.01 g/mL SnCl_2_ solution for about 30 s. The Sn^2+^ ions that remained on the air hole surface shortened the plating time and improved the adhesion between the air hole surface and the silver particles reduced afterwards. Then, a layer of silver film was deposited on the inner surface of the holes. In our experiment, 5% ammoniacal silver solution in an alkaline solution (4% KOH) and 5% glucose solution were used as the plating and reducing agent, respectively. The solutions were then mixed and forced to flow through the air holes of the LSCF by the syringe pump at 5 mL/min flow rate, and the temperature was controlled about 16 °C. The silver-coated LSCF was carefully washed with deionized water and ethanol in succession by the vacuum pump and then blow-dried with nitrogen gas. Furthermore, a three-way valve was employed to switch the fluid path to either a syringe pump or a vacuum pump during the coating process. To characterize the morphology of the fabricated LSCF, a scanning electron microscope (SEM, Zeiss Gemini 300, Jena, Germany) was adopted. The SEM image of the whole cross section of the fabricated silver-coated LSCF is shown in Figure 2b. Then, the morphology of the deposited silver layer in the LSCF is shown in Figure 2c. It can be seen that a uniform silver layer has been coated on the inner surface of LSCF successfully. The average thickness of the silver layer is measured as approximately 46.52 nm. In addition, 10-cm-long pieces of fiber were cut off from the fabricated whole Ag-coated LSCF to be used as the sensor in the follow-up experiments.

### 2.4. Preparation of SERS Sensing Surface

**Figure 3:**
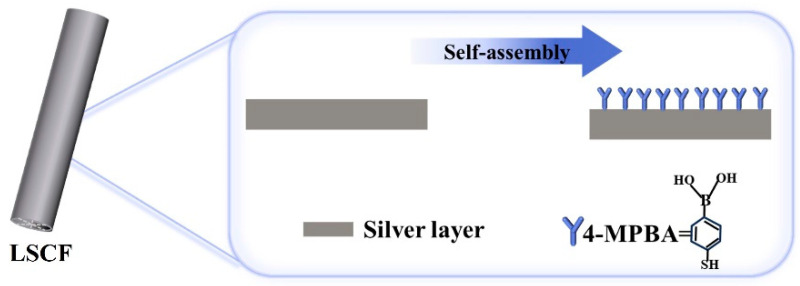
A schematic illustration of the 4-MPBA-modified silver layer.

Because the boronic acid can recognize the cis-diol configuration (e.g., saccharide, glycoprotein, nucleosides, and nucleotides) by forming the reversible complexes, phenylboronic acid and its derivatives have been mostly reported as recognition molecules in many applications of biosensing fields [41,42,43,44]. Therefore, the introduction of 4-mercaptophenylboronic acid (4-MPBA) materials can act as a versatile recognition layer in the optical fiber bio sensors. In this work, the 4-MPBA is also employed and self-assembled on the inner surface of the LSCF. The 4-MPBA monolayer is coated on the silver layer in the LSCF by the SAM technique. The deposition process of the 4-MPBA layer is shown in Figure 3. To cause the molecules to self-assemble onto the silver layer surface, the silver-coated LSCF was immersed in 5 mM 4-MPBA solution that was dissolved in ethanol for 10 h to form the monolayer of 4-MPBA. Then, the LSCF was rinsed with ethanol and deionized water in succession to clear the unbonded molecules. Finally, the LSCF was dried with nitrogen gas. The Raman scattering spectrum of the deposited 4-PMBA was measured in the experiments to evaluate the SERS property of the silver-coated LSCF sensor.

### 2.5. Experimental Setup

**Figure 4:**
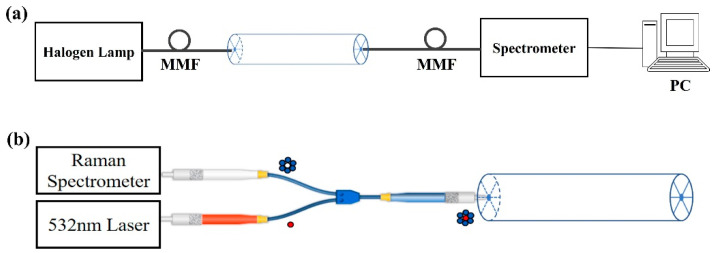
Schematic diagram of the experimental measuring system. (**a**) Measured transmission spectra. (**b**) Measured Raman spectra.

The experimental system for the measurement of the transmission spectra and Raman spectra of the fabricated LSCF is shown in Figure 4. The experimental setup for the transmission spectra measurement is shown in Figure 4a. The light source in the experiment is a halogen lamp. The broadband light beam emitted from a halogen lamp is launched into the 4-MPBA/Ag-coated LSCF sensor via the multimode fiber (MMF). The output light from the sensor is collected via another MMF and detected by the spectrometer (PG2000, Ideaoptics, Shanghai, China). Then, the SPR status in the 4-MPBA/Ag-coated LSCF sensor could be obtained by analyzing the SPR dip in the measured transmission spectrum. The Raman spectra of the 4-MPBA was measured by the experimental setup shown in Figure 4b. The custom-made Y-type optical fiber patch cable (Shanghai Oceanhood opto-electronics tech Co., Ltd., Shanghai, China) consists of the fiber bundles of seven low-fluorescence 105/125 μm MMFs. The seven MMFs are arranged as the pattern shown in Figure 4b, which has one central MMF (red in the figure) and six surrounding MMFs (blue in the figure). The central MMF transmits the excitation light from the 532 nm laser to the solid core of the 4-MPBA/Ag-coated LSCF, while the six surrounding MMFs transmit the backward Raman scattering signal from the six silver-coated air holes to the Raman spectrometer. The all-fiber sensing system eliminates the need for complex and alignment-sensitive free space optical components such as a dichroic mirror and lens. Since the arrangement of the air holes of the LSCF matches the six surrounding fibers, less fluorescence signal from the silica core is collected, leading to a higher signal-to-noise ratio. Compared with the forward collection mode, the backward collection mode provides the convenience of single-ended devices and an improved signal-to-pump ratio [45]. The laser power for the Raman scattering spectral measurements was adjusted to 50 mW in our experiments.

## 3. Results and Discussion

**Figure 5:**
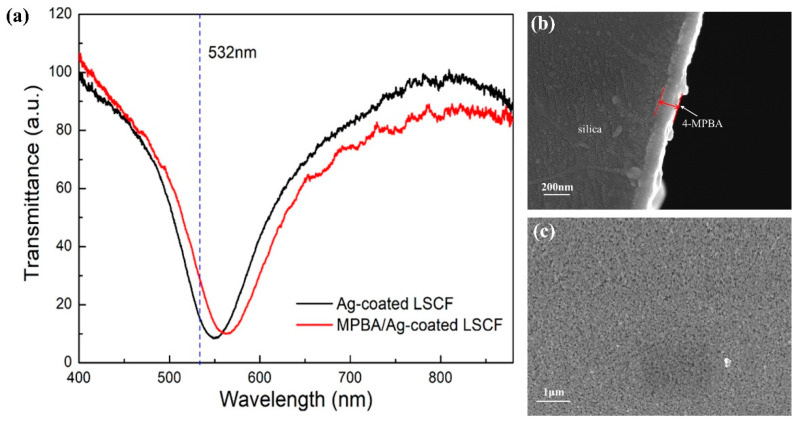
(**a**) Transmittance spectra of the LSCF before (black line) and after (red line) 4-MPBA modification. (**b**) SEM images of the cross section of the 4-MPBA monolayer on the surface. (**c**) Surface SEM morphology of 4-MPBA/Ag-coated LSCF.

To realize the Raman scattering spectrum detection, the modification of 4-MPBA on the LSCF surface is of great importance to the experiment. The SPR spectra of the fiber sensor were measured before and after the SAM of 4-MPBA to evaluate the surface modification. In the measurement, the air holes of LSCF were filled with deionized water with an RI of 1.3326. As shown in Figure 5a, the red and black solid curves represent the SPR spectra of the silver-coated LSCF with and without an additional 4-MPBA layer, respectively. It can be seen that the resonance wavelength of LSCF undergoes a 15 nm red-shift after 4-MPBA modification. This result indicates that the 4-MPBA was immobilized on the sensing surface by the formation of Ag-S bond [43,46]. The dashed line represents the 532 nm laser adopted in the Raman scattering experiments. It can be seen that the laser wavelength is located appropriately in the SPR dip. Therefore, when the 532 nm laser is transmitted through the LSCF core, the SPR phenomenon will be excited. Then, the electromagnetic field around the 4-MPBA layer is enhanced, resulting in the enhancement of the Raman scattering signal strength. For further analysis, the 4-MPBA monolayer on the inner surface of LSCF was also characterized by SEM. Figure 5b shows the cross-sectional SEM images of 4-MPBA layer on the surface. A surface morphology SEM image of 4-MPBA/Ag-coated LSCF is displayed in Figure 5c. The relatively uniform 4-MPBA monolayer can be seen clearly in the SEM photos, which also proves the successful deposition of the 4-MPBA layer in the silver-coated LSCF.

**Figure 6:**
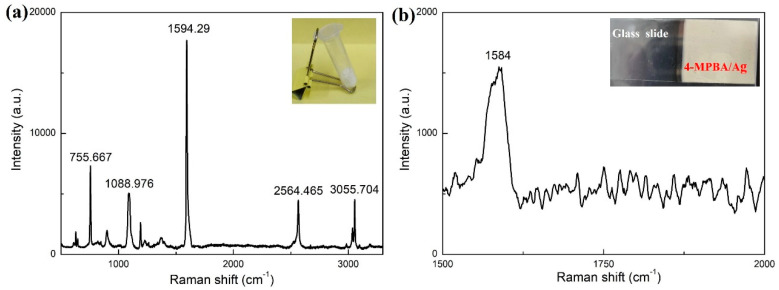
Raman spectrum of (**a**) 4-MPBA powder, (**b**) 4-MPBA monolayer adsorbed on the silver layer. Insets in (**a**,**b**): Photo of 4-MPBA powder and 4-MPBA/Ag-coated glass slide, respectively.

The Raman scattering spectrum of the 4-MPBA powder was firstly measured for reference, which is shown in Figure 6a. The inset is the photo of the 4-MPBA powder. There are several characteristic peaks in the Raman scattering spectrum, including 755.667 cm^−1^, 1088.976 cm^−1^, 1594.29 cm^−1^, 2564.465 cm^−1^, and 3055.704 cm^−1^. The peak at 1594.29cm^−1^ possesses the strongest signal strength, which is the optimal choice for quantitative analysis. It is to be noted that the Raman scattering spectrum of the 4-MPBA may vary slightly when it assembles on the surface of the silver. Therefore, the Raman scattering spectrum of the self-assembled PMBA monolayer on the silver-coated glass slide was also measured to be compared with the Raman spectrum of the 4-MPBA powder. The Raman spectrum is shown in Figure 6b. The exposure time is set as 20 min due to the weak signal strength. In comparison to the normal Raman spectrum of 4-MPBA powder, the strong Raman peak at 1594.29 cm^−1^ due to the S-H stretching vibration of 4-MPBA shifts to 1584 cm^−1^ in the Raman spectrum of the 4-MPBA monolayer on the silver surface, which reflects that 4-MPBA is stably adsorbing on the surface of the silver film via the thiol group [46,47]. The photo of the 4-MPBA/Ag-coated glass slide is shown in the inset of Figure 6b. The brief fabrication process of the 4-MPBA/Ag-coated glass slide is as follows: The first step is a layer of silver film that is deposited on the surface of the glass slide. It is achieved by using the silver mirror reaction. The silver mirror reaction is achieved by using the silver nitrate solution and the glucose solution on the glass slide substrate. The next step is to coat a 4-MPBA monolayer on the sensing surface by the self-assembled method. The silver-coated glass slide is immersed in 5 mM 4-MPBA ethanolic solution for 10 h to form a 4-MPBA monolayer, and then rinsed successively with deionized water and ethanol.

**Figure 7:**
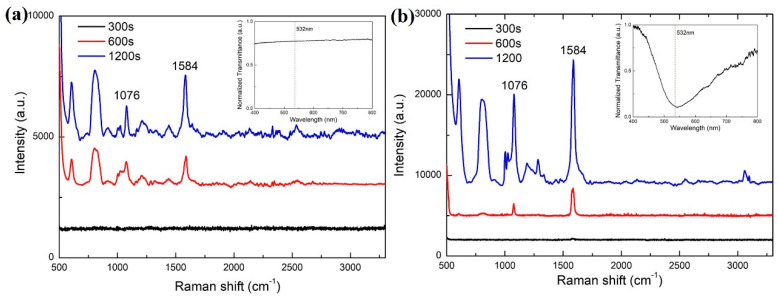
Raman spectra of the 4-MPBA/Ag-coated LSCF filled (**a**) with air (non-enhanced case) and (**b**) with deionized water (surface-enhanced case). Insets in (**a**,**b**): Transmittance spectra of the 4-MPBA/Ag-coated LSCF filled with air and deionized water, respectively (dashed line represents the 532 nm laser).

To demonstrate the SERS sensing ability of the proposed LSCF sensor, the Raman scattering spectra of the 4-MPBA/Ag-coated LSCF under the non-enhanced and SPR enhanced cases were both measured with different exposure time for comparison. The Raman scattering spectra of the 4-MPBA/Ag-coated LSCF under the non-enhanced case is shown in Figure 7a. Here, the air holes of the LSCF are filled with air. In such case, there is no SPR dip in the transmission spectrum as shown in the inset of Figure 7a, which indicates that the SPR phenomenon cannot be excited by the 532 nm laser. Thus, the Raman scattering signal is not enhanced. Two main characteristic Raman peaks of the 4-MPBA monolayer are located at 1076 cm^−1^ and 1584 cm^−1^, respectively. The strength of Raman scattering peaks increases with the exposure time. When the exposure time is 1200 s, the intensities of the characteristic Raman peaks at 1076 cm^−1^ and 1584 cm^−1^ are 1300 and 2450, respectively. The Raman spectra of the 4-MPBA/Ag-coated LSCF under the surface enhanced case are shown in Figure 7b. In this case, the air holes of the LSCF are filled with deionized water. As shown by the transmission spectrum in the inset of Figure 7b, the SPR phenomenon is excited. The SPR dip is located around 532 nm, which is indicated by dashed line in the inset. Therefore, when the 532 nm laser is transmitted through the solid core of LSCF, the SPR phenomenon is excited, and an electromagnetic field enhancement occurs at the surface of the silver layer where the 4-MPBA monolayer is located. Then, the signal strength of the Raman scattering is enhanced. The strength of Raman scattering peaks also increases with the exposure time. In the Raman scattering spectrum for the exposure time of 1200 s in Figure 7b, the intensities of the characteristic Raman peaks at 1076 cm^−1^ and 1584 cm^−1^ are 11,300 and 15,400, respectively. Compared with the non-enhanced case shown in Figure 7a, the Raman scattering signal strength of peaks at 1076 cm^−1^ and 1584 cm^−1^ were enhanced 8.7 and 6.3 times, respectively. These results indicate that the Raman scattering signal of the 4-MPBA assembled on the silver surface in the silver-coated LSCF was successfully enhanced by the SPR phenomenon excited in the LSCF. Therefore, the proposed silver-coated LSCF sensor possesses the capability of SERS sensing.

## 4. Conclusions

In conclusion, we developed an LSCF SERS probe based on a silver-coated large-core suspended-core fiber for SERS sensing. A thin silver layer is coated homogeneously along the length of the fiber on the inner surface of the air holes utilizing the liquid-phase deposition method. Meanwhile, the fragile silver layer is protected inside the LSCF from contamination and corrosion. Then, 4-MPBA is adsorbed on the silver layer by SAM technique. The RW can be adjusted to around the excitation wavelength of Raman scattering by filling the air holes with liquid with appropriate RI. When the laser is transmitted in the solid core, an enhancement of the electromagnetic field at the surface of the analyte occurs due to the excitation of SPP. The SERS signal is propagated in the silver-coated air channels instead of the silica core and collected by the Y-type optical fiber patch cable, which can effectively reduce the Raman and fluorescence background of the silica core. The long interaction length between the light and analyte enables the SERS signal to be accumulated along the entire fiber length. Experiments were taken to measure the Raman spectra of the 4-MPBA/Ag-coated LSCF under the non-enhanced and surface enhanced case, respectively. The result shows that the Raman signal strength is enhanced more than 6 times compared with the non-enhanced case. Moreover, an accumulative effect of SERS signal along the SERS-active fiber length is demonstrated experimentally. The combination of SERS and the micro-structured optical fibers offers a potential approach for SERS detection. This work will find the potential applications in glucose, cholesterol detection, and other biomedical areas.

## Data Availability

Data are contained within the article.
